# GP-Driven Adaptive Tube MPC for Communication-Preserving Navigation of Mobile Relay Robots in Indoor Disaster Environments

**DOI:** 10.3390/s26133981

**Published:** 2026-06-23

**Authors:** Dongju Kim, Sungjae Kim, Jin-Ho Suh

**Affiliations:** 1 Department of Intelligent Robot Engineering, Pukyong National University, Busan 48513, Republic of Korea; sanbba2214@pukyong.ac.kr; 2 The Industrial Science Technology Research Center, Pukyong National University, Busan 48513, Republic of Korea; bbman7020@gmail.com; 3 Major of Mechanical System Engineering, Pukyong National University, Busan 48513, Republic of Korea

**Keywords:** mobile relay robots, Gaussian process regression, adaptive tube MPC, communication-preserving navigation, indoor disaster environments

## Abstract

**Highlights:**

**What are the main findings?**

- A communication-aware MPC framework is developed that combines Gaussian process regression (GPR)-based spatial RSSI prediction with adaptive tube tightening for mobile relay navigation in indoor NLOS environments.
- A velocity- and uncertainty-adaptive ellipsoidal error tube is introduced to tighten both communication and obstacle constraints over the full closed-loop deviation set, enabling more robust relay motion than nominal or heuristic-margin communication-aware control.

**What are the implications of the main findings?**

- Across two indoor environments and 50 Monte Carlo runs each, the proposed method attains the highest connectivity satisfaction rate among controllers that preserve a safe motion margin, with significantly fewer connectivity violations than nominal and heuristic adaptive-margin MPC; a reactive connectivity-first baseline reaches marginally higher raw connectivity only at three to four times the near-collision rate.
- The proposed framework improves the practical balance among communication preservation, collision avoidance, and forward mission execution while maintaining millisecond-level online solve times.

**Abstract:**

Maintaining reliable communication while ensuring collision-free motion is a central challenge for mobile relay robots operating in indoor disaster environments, where abrupt non-line-of-sight (NLOS) degradation and narrow structural bottlenecks can severely disrupt multi-hop connectivity. To address this problem, this paper proposes a Gaussian Process-Driven Adaptive Tube Model Predictive Control (GP-ATMPC) framework for communication-preserving relay navigation. Gaussian process regression (GPR) is used to construct a probabilistic spatial radio map from sparse received signal strength indicator (RSSI) measurements, providing both the predicted channel mean and its uncertainty over unvisited regions. Motion uncertainty is represented by an adaptive ellipsoidal error tube whose radius varies with translational motion, angular motion, and localization uncertainty. Based on this tube model, both obstacle and communication constraints are tightened over the full closed-loop state tube via a tube-tightened lower confidence bound (LCB) that jointly accounts for radio-prediction and motion-tracking uncertainty. Across two indoor disaster environments and 50 Monte Carlo runs each, the proposed method attains the highest connectivity satisfaction rate among controllers that preserve a safe motion margin, with significantly fewer end-to-end connectivity violations than nominal and heuristic adaptive-margin MPC by a paired Wilcoxon test, while maintaining millisecond-level online solve times. A reactive connectivity-first baseline reaches slightly higher raw connectivity but at three to four times the near-collision rate and without feasibility or stability guarantees. The radio-prediction layer is further validated in a higher-fidelity Gazebo environment and on real indoor RSSI measurements, where it reconstructs the measured channel with a mean absolute error of about 2.1 dB. These results indicate that coupling spatial radio prediction with adaptive tube-based robust control provides an effective framework for resilient communication-aware relay navigation in degraded indoor environments.

## 1. Introduction

Reliable wireless communication is indispensable for autonomous multi-robot operation in indoor disaster environments, where damaged infrastructure, collapsed structures, and power outages frequently disable conventional networking services. In such settings, mobile relay robots must dynamically establish and maintain a multi-hop communication backbone to support forward exploration, victim search, and remote situational awareness. This problem has been widely recognized in search-and-rescue robotics, communication-aware robotics, and robotic wireless sensor networks, where communication is treated not as a background assumption but as a mission-critical resource directly affecting exploration performance and operational safety [1,2,3].

Communication-aware relay deployment and motion planning have shown that robot mobility can actively improve network performance [4,5,6,7,8,9,10]. However, most existing approaches rely on deterministic or reactive channel models and do not explicitly represent predictive spatial uncertainty in previously unvisited indoor regions.

Connectivity-preserving control and relay-placement strategies have also been studied to maintain network topology under multi-robot operation [11,12,13,14,15,16,17,18,19]. Beyond such topology-level approaches, GPR-based radio mapping is highly relevant to this problem because it can estimate both the expected RSSI field and its uncertainty from sparse measurements [20,21,22,23,24,25,26,27]. Nevertheless, most prior studies are limited to the perception layer and do not directly embed radio uncertainty into a robust receding-horizon controller for communication-preserving relay navigation.

Uncertainty analysis and robust control of robotic systems have also been advanced from complementary directions, including model-based virtual sensing that infers unobservable states and propagates measurement uncertainty in cooperative robots [28], visual–tactile fusion for accurate coordinate-system alignment of mobile robots under environmental variation [29], and Bayesian reliability and stability analysis of robotic motion systems under multi-source uncertainty [30]. These works reinforce the broader need to model and propagate uncertainty explicitly in robotic systems, which the present work addresses by jointly handling radio prediction and motion uncertainty within a robust tube-based controller.

Robust and tube-based MPC provide complementary tools for enforcing constraints under bounded disturbances [31,32,33,34,35,36,37,38,39], but standard formulations do not directly capture the joint effect of radio-map uncertainty and motion-tracking uncertainty. In particular, they do not inherently provide a communication-preserving condition that remains valid over the full closed-loop state tube when the future RSSI field is uncertain and learned online [40].

Our previous ARX-KF-based relay-deployment study [41] addressed part of this challenge by adapting to temporal channel residuals and maintaining connectivity under time-varying environmental variation. Its limitation, however, was that it remained fundamentally reactive because it updated the channel estimate from already visited trajectories and could not explicitly infer the spatial structure of unvisited radio shadowing. The improvement is therefore threefold: spatial inference with calibrated uncertainty over unvisited regions replaces the purely temporal estimate, the communication and obstacle constraints are tightened over the full closed-loop deviation tube rather than evaluated at the nominal point, and the resulting controller carries the recursive feasibility and practical-stability guarantees established in Section 4.5. This paper addresses that gap by combining residual-based GPR radio field prediction with adaptive tube MPC so that communication and obstacle constraints are tightened over the full closed-loop deviation tube rather than only along the nominal trajectory. In particular, a tube-tightened lower confidence bound is derived by combining the GPR-based radio estimate with a local smoothness bound of the communication field, thereby accounting simultaneously for radio-prediction uncertainty and motion-tracking uncertainty.

The main contributions of this paper are summarized as follows.

- A residual-based GPR radio map that predicts the spatial RSSI mean and its uncertainty over unvisited regions, enabling proactive avoidance of communication-risk areas rather than reactive adaptation along previously visited trajectories.
- An adaptive ellipsoidal tube MPC for differential-drive relays whose size adapts to translational velocity, angular motion, and localization uncertainty, replacing fixed heuristic safety margins with an explicitly propagated closed-loop uncertainty set.
- A tube-tightened communication constraint is derived by combining the GPR-based lower confidence bound with a local smoothness bound of the radio field. This yields a sufficient model-based condition for communication preservation over the closed-loop tube, thereby accounting simultaneously for radio-prediction uncertainty and motion-tracking error.
- Validation of the unified framework across two indoor disaster environments with 50 Monte Carlo runs each, a higher-fidelity Gazebo study, and real indoor RSSI measurements.

The remainder of this paper is organized as follows. Section 2 introduces the relay-network architecture, the differential-drive motion model, and the nominal indoor radio model, and formulates the communication-preserving navigation problem. Section 3 develops the GPR-based spatial radio-shadowing map, including the residual model, posterior prediction, and local smoothness bound. Section 4 presents the proposed adaptive tube MPC, in which both communication and obstacle constraints are tightened over the closed-loop tube. Section 5 reports the component-wise ablation, the 50-run Monte Carlo comparisons in the Three-Room and Warehouse environments, the Gazebo validation, and the real indoor RSSI validation. Section 6 concludes the paper.

## 2. System Model and Problem Formulation

This section presents the relay-network architecture, the differential-drive motion model of each mobile relay robot, and the nominal indoor radio model used to separate large-scale path loss from spatial shadowing residuals. Based on these components, the communication-preserving relay-navigation problem is formulated as a constrained finite-horizon optimal control problem, which will later be reformulated in Section 4 as an adaptive tube MPC under spatial radio uncertainty.

### 2.1. Relay-Network Architecture and Local Planning View

We consider an indoor disaster environment in which a stationary base station communicates with a forward exploration agent through a set of mobile relay robots. The relays are responsible for maintaining a communication backbone while repositioning themselves according to the motion of the exploration front and the local radio environment. Let the set of all agents be denoted by $A=\{b,e,r_{1},\ldots ,r_{N_{R}}\},$ where b is the base station, e is the forward explorer, and $r_{1},\ldots ,r_{N_{R}}$ are the mobile relays. At each control step k, each relay i is assigned a parent node $\pi_{i}(k)\in A\setminus \{i\}$, which may be the base station, another relay, or the explorer, and with which the relay must preserve a reliable communication link.

The controller is derived from a local viewpoint: a single relay plans its motion over the prediction horizon while parent-link assignment and parent motion are treated as exogenous inputs from a higher network layer. This local viewpoint isolates the communication-preserving navigation mechanism that can later be instantiated independently at multiple relays. Let $p_{k}$ denote the planar position of the parent node at step k, and let $z_{k}$ denote the planar position of the relay. The objective of the relay is to move through the obstacle-populated workspace while preserving the parent link and positioning itself so as to support the downstream communication chain.

### 2.2. Differential-Drive Relay Model and Nominal Radio Representation

Each mobile relay is modeled as a differential-drive robot evolving on a planar workspace [34]. The relay state and control at step k are defined in Equation (1) as

(1) $$s_{k}=[x_{k},\;\;y_{k},\;\;\theta_{k}{]}^{⊤}\in R^{3},u_{k}=[v_{k},\;\;\omega_{k}{]}^{⊤}\in R^{2}.$$

In Equation (1), $\left(x_{k},y_{k}\right)$ denotes the Cartesian position of the relay, $\theta_{k}$ is the heading angle, and $v_{k},\omega_{k}$ are the translational and angular velocities. The planar position is denoted $z_{k}=[x_{k},y_{k}{]}^{⊤}\in R^{2}$. Using forward Euler discretization with sampling time $T_{s}>0$, and including a bounded disturbance $w_{k}$ that captures actuation mismatch, wheel slip, localization drift, and local linearization residual, the relay kinematics can be written as Equation (2):

(2) $$s_{k+1}=f(s_{k},u_{k})+w_{k}=s_{k}+T_{s}\left[\begin{array}{c} v_{k}\cos\theta_{k}\\ v_{k}\sin\theta_{k}\\ \omega_{k} \end{array}\right]+w_{k}.$$

The admissible input set and obstacle-free workspace constraints are given by $u_{k}\in U=\left\{u=[v,\omega {]}^{⊤}:|v|\leq v_{max},\;\;|\omega |\leq \omega_{max}\right\},z_{k}\in Z_{free}\subset R^{2}.$ Because the relay is non-holonomic, collision avoidance and communication recovery cannot be achieved through arbitrary lateral displacement. Instead, they must be realized through coupled forward motion and heading change. This motivates the anisotropic and state-dependent tube construction developed later in Section 4.

Communication with the parent node is modeled as the sum of a nominal distance-dependent term and an unknown spatial shadowing residual. Let the Euclidean communication distance be defined as $d(z,p)=∥z-p{∥}_{2}$. Then, the received signal strength indicator (RSSI), expressed in dBm, is modeled in Equation (3) as

(3) $$r(z,p)=r_{nom}(d(z,p))+\delta (z,p)+\varepsilon ,$$

where $r_{nom}(\cdot )$ is the nominal propagation model, δ(z,p) is the unknown spatial shadowing residual associated with the parent–relay link, and ε is measurement noise.

The nominal radio model is then given by Equation (4):

(4) $$r_{nom}(d)=r_{0}-10n{log}_{10}\left(\frac{d}{d_{0}}\right)-L_{env},$$

where $r_{0}$ is the reference received power at a nominal distance $d_{0}$, n is the path-loss exponent, and $L_{env}$ captures any deterministic attenuation associated with coarse environmental structure [42,43].

In this representation, the residual term δ(z,p) describes unmodeled NLOS attenuation induced by walls, corners, doorways, and bottlenecks, while ε accounts for measurement noise. Rather than learning absolute RSSI directly, the proposed framework models the residual after removing the nominal distance-dependent trend. This residual becomes the learning target in Section 3. Because the parent trajectory is treated as known over one MPC horizon, the communication model can be evaluated for each candidate relay state together with the corresponding parent state at that step, which makes the relay-navigation problem well suited to local receding-horizon optimization with externally supplied parent motion.

### 2.3. Communication-Preserving Navigation Objective

At every sampling instant, the relay computes a finite-horizon control sequence that moves it toward a reference support position $z_{j|k}^{ref}$ while preserving communication with its parent node and avoiding collisions. The generic communication-preserving navigation problem can therefore be formulated as the finite-horizon optimal control problem in Equation (5):

(5) $$\underset{\left\{u_{j|k}\right\}}{\min}\sum_{j=0}^{N_{p}-1}\left({\left\|z_{j|k}-z_{j|k}^{ref}\right\|}_{Q}^{2}+{\left\|u_{j|k}\right\|}_{R}^{2}\right)\;s.\;t.\;{\;s}_{j+1|k}=f(s_{j|k},u_{j|k})+w_{j|k},\;\;r(z_{j|k},p_{j|k})\geq r_{min},\;\;z_{j|k}\in Z_{free},\;\;u_{j|k}\in U.$$

In Equation (5), $r_{min}$ denotes the minimum RSSI threshold required for operational communication continuity, $N_{p}$ denotes the prediction horizon, and all constraints hold for $j=0,\ldots ,N_{p}-1$.

In practice, however, the nominal problem in Equation (5) cannot be enforced directly. The obstacle and communication constraints depend on the actual future relay state, whereas the optimizer reasons only over a nominal predicted trajectory. In addition, the communication constraint depends on an unknown residual field evaluated at future, potentially unvisited locations. Therefore, both motion uncertainty and spatial radio uncertainty must be accounted for explicitly. This motivates the two-layer design adopted in the remainder of the paper: Section 3 constructs a probabilistic spatial radio model, and Section 4 embeds that model into a robust tube-based MPC so that both communication and physical-safety constraints are evaluated conservatively over the admissible closed-loop tube rather than only at the nominal state.

## 3. Spatial Radio-Shadowing Mapping via GPR

As introduced in Section 2, communication preservation cannot be enforced reliably from a deterministic path-loss model alone, because previously unvisited indoor regions may exhibit abrupt NLOS attenuation. To address this limitation, this section develops a GPR-based spatial radio map that estimates both the expected RSSI and its predictive uncertainty over unexplored regions. The resulting radio layer provides the mean field, the uncertainty field, the LCB, and a local smoothness bound that will later be used in the adaptive tube MPC of Section 4.

### 3.1. Residual-Based Dataset and Gaussian-Process Model

Each relay continuously records RSSI measurements from its assigned parent node together with its own planar position and the corresponding parent position. The retained local radio dataset at step k can therefore be written as Equation (6):

(6) $$\;\;\;\;\;\;\;\;\;\;D_{k}=\{(z_{i},p_{i},y_{i}){\}}_{i=1}^{N_{k}},$$

where $z_{i}$ denotes the relay position of the i-th observation, $p_{i}$ denotes the associated parent position, and $y_{i}=r_{meas,i}-r_{nom}(d(z_{i},p_{i}))$ is the residual observation obtained after removing the nominal distance-dependent trend. By construction, the residual observation satisfies $y_{i}=\delta (z_{i},p_{i})+\varepsilon_{i},$ which means that the learning layer focuses on spatially correlated shadowing rather than relearning the large-scale path-loss component. For notational economy, we write δ(q) with $q\in R^{2}$ denoting the query relay position; the corresponding parent position is treated as exogenous over the prediction horizon and is omitted from the argument list whenever it is unambiguous.

Accordingly, the residual field is modeled as a Gaussian process, and the prior together with the covariance function is defined in Equation (7) as

(7) $$\delta (q)∼GP(0,k(q,q^{\prime })),k(q,q^{\prime })=\sigma_{f}^{2}exp(-\frac{∥q-q^{\prime }{∥}_{2}^{2}}{2l^{2}}),$$

where $\sigma_{f}$ is the signal amplitude and l is the characteristic spatial length scale. The zero-mean prior in Equation (7) is appropriate because the nominal propagation model in Equation (4) already accounts for the large-scale mean trend. The squared-exponential kernel is particularly convenient here because it yields a smooth posterior field and analytically differentiable expressions, which will later be used to construct a communication-smoothness bound in Section 3.3. The hyperparameters $\theta_{GP}=\{\sigma_{f},l,\sigma_{n}\}$, where $\sigma_{n}$ is the measurement noise standard deviation is estimated by marginal-likelihood maximization over the retained dataset [44].

The configured values in Table 1 are physically grounded. The signal amplitude $\sigma_{f}$ (about 5 dB) matches the depth of the NLOS shadowing residual at doorways and corners; the length scale l (on the order of 1.0 to 1.5 m) matches the spatial scale of indoor structures such as door frames and wall edges; and the noise term $\sigma_{n}$ (1.5 dB) matches the RSSI measurement noise. The confidence coefficient β in Equation (10) is set to 1.2, a one-sided multiplier that penalizes high-uncertainty regions without over-tightening the connectivity constraint. That this choice is not specific to the simulation is confirmed in Section 5.6, where the length scale fitted on real indoor RSSI, about 0.9 to 1.5 m, agrees with the configured value.

To preserve online tractability, the GP memory is kept bounded in implementation. Each link-local GP maintains at most $N_{max}=70$ samples. The most recent $N_{\mathrm{rec}}=25$ samples are always retained, and the remaining entries are selected by nearest-neighbor pruning in the normalized link-geometry space, with up to $N_{\mathrm{near}}=30$ spatially relevant samples retained. The GP hyperparameters are initialized with $\sigma_{f}=5$, $\sigma_{n}=1.5$ dB, and length scales in the range of 1.0–1.5 m, and are re-estimated periodically by marginal-likelihood maximization when sufficient new samples are available, rather than at every MPC step. When the parent assignment changes, the corresponding link-local buffer is reinitialized to avoid mixing measurements from different link geometries.

In the implementation, the GP model is maintained link-locally for each assigned parent–relay pair over the current planning window. Therefore, although the parent position is omitted from the compact notation δ(q), the retained dataset and posterior prediction are conditioned on the current parent-link context. When the parent assignment changes, the radio buffer is either reinitialized or queried using samples associated with the corresponding link context.

### 3.2. Posterior Prediction of the Spatial RSSI Field

Given the retained dataset in Equation (6), the GP posterior is evaluated at a query relay position q. Let the kernel Gram matrix be defined by $\left[K{]}_{ij}=k(z_{i},z_{j}\right)$, and let the cross-covariance vector be $k_{q}=[k(q,z_{1}),\ldots ,k(q,z_{N_{k}}){]}^{⊤}$. Then the posterior mean of the latent residual is obtained in Equation (8) as

(8) $$\mu_{\delta }(q)=k_{q}^{⊤}(K+\sigma_{n}^{2}I{)}^{-1}y,$$

where $y=[y_{1},\ldots ,y_{N_{k}}{]}^{⊤}$ is the stacked residual-observation vector. Similarly, the posterior variance of the latent residual is given in Equation (9) as

(9) $$\sigma_{\delta }^{2}(q)=k(q,q)-k_{q}^{⊤}(K+\sigma_{n}^{2}I{)}^{-1}k_{q}.$$

Equations (8) and (9) characterize the posterior distribution $\delta (q)|D_{k}∼N(\mu_{\delta }(q),\sigma_{\delta }^{2}(q))$. In this posterior model, the mean term in Equation (8) estimates the expected shadowing residual, whereas the variance term in Equation (9) quantifies epistemic uncertainty due to incomplete spatial sampling. If the query point lies near previously traversed paths, the covariance with the retained dataset remains strong, and the posterior variance decreases. In contrast, if the query point lies deep inside an unvisited room or behind an unseen corner, the covariance weakens and the posterior variance increases toward its prior level. In this way, the GPR naturally indicates regions where the communication forecast is less reliable.

The residual prediction is then recombined with the nominal propagation model in Equation (4) to obtain the absolute RSSI field. Specifically, the mean RSSI field and its predictive uncertainty are written as $\mu_{r}(q)=r_{nom}(d(q,p))+\mu_{\delta }(q),\sigma_{r}(q)=\sigma_{\delta }(q)$, where the parent position p over the prediction horizon is supplied by the higher network layer.

### 3.3. Conservative Communication Field and Local Smoothness Bound

Using only the mean prediction $\mu_{r}\left(q\right)$ is unsafe in cluttered disaster environments because a location with acceptable expected signal strength may still exhibit substantial predictive uncertainty. To incorporate this uncertainty into control, the conservative communication estimate is defined by the lower confidence bound in Equation (10):

(10) $$LCB(q)=\mu_{r}(q)-\beta \sigma_{r}(q),\beta >0,$$

where β is a confidence-scaling coefficient. Equation (10) shows that poorly explored regions are penalized automatically: when the uncertainty increases, the conservative communication estimate decreases even if the mean RSSI remains moderate. As a result, doorways, corners, and shadowed corridor segments can be treated as communication-risk areas before the relay physically enters them.

Based on Equation (10), the nominal communication-feasible set may be interpreted as $\left\{q\in R^{2}:LCB(q)\geq r_{min}\right\}$, but this nominal set alone is not sufficient for robust control because the actual robot state may deviate from the nominal prediction used inside the optimizer. Therefore, the controller also requires a local bound on the spatial variation in the conservative communication field.

Because the squared-exponential kernel in Equation (7) is smooth, the LCB field is locally Lipschitz on compact neighborhoods [44]. The local smoothness bound around a nominal planar position ${\bar{z}}_{k}$ is therefore defined in Equation (11) as

(11) $$L_{k}=\underset{z\in B({\bar{z}}_{k},b_{k})}{sup}∥\nabla LCB(z){∥}_{2},$$

where $B({\bar{z}}_{k},b_{k})$ denotes the closed ball of radius $b_{k}$ centered at ${\bar{z}}_{k}$. Using the local bound in Equation (11), the variation in the conservative communication field around the nominal position can be bounded by Equation (12):

(12) $$LCB(z)\geq LCB({\bar{z}}_{k})-L_{k}∥z-{\bar{z}}_{k}{∥}_{2},z\in B({\bar{z}}_{k},b_{k}).$$

Equation (12) provides the key bridge from radio prediction to the adaptive tube MPC. If the actual relay may deviate from the nominal predicted position by at most $b_{k}$, then the worst-case degradation of the conservative communication field around the nominal point is bounded by $L_{k}b_{k}$. For online implementation, the exact bound in Equation (11) is approximated pointwise at the nominal query point. This practical approximation is written in Equation (13) as

(13) $${\hat{L}}_{k}\approx \kappa (∥\nabla \mu_{r}({\bar{z}}_{k}){∥}_{2}+\beta ∥\nabla \sigma_{r}({\bar{z}}_{k}){∥}_{2}),\;\;\;\;\;\;\;\kappa \geq 1.$$

Equation (13) is used online as a computationally efficient surrogate for the neighborhood supremum in Equation (11), and a brief argument shows that, with a mild inflation, it upper-bounds the exact local bound rather than merely sampling it. The gradient of the lower confidence bound is first bounded by the triangle inequality as ${∥\nabla LCB\left(z\right)∥}_{2}={∥\nabla \mu_{r}\left(z\right)-\beta \;\nabla \sigma_{r}\left(z\right)∥}_{2}\leq {∥\nabla \mu_{r}\left(z\right)∥}_{2}+\beta \;{∥\nabla \sigma_{r}\left(z\right)∥}_{2}$, so any cancellation between the mean and uncertainty gradients is discarded, and the right-hand side overestimates the true gradient at every point. To then cover the gap between the pointwise value at the nominal point and the neighborhood supremum, the smoothness of the squared-exponential kernel in Equation (7) is used: the posterior mean and standard deviation are infinitely differentiable, and their Hessian spectral norm on the compact ball $B\left({\bar{z}}_{k},b_{k}\right)$ is bounded in closed form by a constant $H_{k}$ that depends only on the kernel signal variance and length scale, so a first-order Taylor argument gives ${∥\nabla LCB\left(z\right)∥}_{2}\leq {∥\nabla LCB\left({\bar{z}}_{k}\right)∥}_{2}+H_{k}b_{k}$ for all $z\in B\left({\bar{z}}_{k},b_{k}\right)$.

Combining the two relations, the exact local bound in Equation (11) satisfies $L_{k}\leq {∥\nabla \mu_{r}\left({\bar{z}}_{k}\right)∥}_{2}+\beta \;{∥\nabla \sigma_{r}\left({\bar{z}}_{k}\right)∥}_{2}+H_{k}\;b_{k}\leq \kappa \left({∥\nabla \mu_{r}\left({\bar{z}}_{k}\right)∥}_{2}+\beta \;{∥\nabla \sigma_{r}\left({\bar{z}}_{k}\right)∥}_{2}\right)$ for an inflation factor κ≥1 calibrated offline. Because κ≥1, the inflated surrogate overestimates the true smoothness bound, so the tube-tightened communication constraint in Equation (23) subtracts at least as much margin as the exact bound would, and feasibility of the approximate constraint implies satisfaction of the exact constraint. The approximation therefore errs on the safe side, trading a small amount of additional conservatism for an order-one per-step gradient evaluation in place of the neighborhood optimization in Equation (11). The radio-mapping layer therefore passes four quantities to the controller at each planning step: the mean RSSI prediction $\mu_{r}$, the predictive uncertainty $\sigma_{r}$, the lower confidence bound defined in Equation (10), and the local smoothness approximation given in Equation (13). These quantities form the minimum probabilistic ingredients needed for the tube-tightened communication constraint developed in Section 4.

## 4. Gaussian Process-Driven Adaptive Tube MPC

Using the probabilistic radio field constructed in Section 3, we now formulate the local communication-preserving controller. The proposed adaptive tube MPC accounts jointly for radio-prediction uncertainty and motion uncertainty by tightening both communication and obstacle constraints over the full closed-loop deviation tube. Throughout this section, the parent-node trajectory over the prediction horizon is treated as known and is supplied by the higher network layer.

### 4.1. Nominal and Actual Relay Dynamics

For receding-horizon control, the disturbed nonlinear model in Equation (2) is locally linearized around a nominal state–input pair $\left({\hat{s}}_{k},{\bar{u}}_{k}\right)$. The resulting actual and nominal linearized dynamics are written together in Equation (14) as

(14) $$s_{k+1}\approx A_{k}s_{k}+B_{k}u_{k}+c_{k}+w_{k},{\hat{s}}_{k+1}=A_{k}{\hat{s}}_{k}+B_{k}{\bar{u}}_{k}+c_{k},$$

where $A_{k}$, $B_{k}$, and $c_{k}$ are obtained from the first-order linearization of the nonlinear relay dynamics. Based on Equation (14), the deviation between the actual and nominal states is defined by the tracking error ${\tilde{e}}_{k}=s_{k}-{\hat{s}}_{k}.$ To stabilize this deviation, the actual control input is chosen according to the affine feedback law given in Equation (15):

(15) $$u_{k}={\bar{u}}_{k}+K_{k}{\tilde{e}}_{k},$$

where $K_{k}$ is an ancillary feedback gain, for example, obtained from a local discrete-time linear–quadratic regulator (LQR) design on the linearized model. Substituting Equation (15) into the difference between the two relations in Equation (14), we obtain the closed-loop error dynamics in Equation (16):

(16) $${\tilde{e}}_{k+1}=(A_{k}+B_{k}K_{k}){\tilde{e}}_{k}+w_{k}.$$

Equation (16) provides the basis for the tube construction developed in the next subsection. In practice, the feedback gain is recomputed online, and a small regularization threshold is used near zero translational speed to avoid ill-conditioned gain computation in narrow passages or near stopping conditions.

### 4.2. Adaptive Ellipsoidal Tube Propagation

To represent the admissible closed-loop deviation around the nominal trajectory, the tracking-error set is modeled as an ellipsoidal tube. This tube is defined in Equation (17) as

(17) $$E_{k}=\{e\in R^{3}:e^{⊤}P^{-1}e\leq \rho_{k}^{2}\},$$

where P≻0 is a fixed shape matrix that determines the anisotropic geometry of the deviation set, and $\rho_{k}>0$ is a time-varying tube radius (in the P-metric) that determines its instantaneous size.

The disturbance is assumed to belong to a bounded ellipsoidal set $W_{k}=\{w:w^{⊤}S_{k}^{-1}w\leq 1\}$, where $S_{k}≻0$ is the disturbance-shape matrix. To reflect the fact that motion uncertainty increases under aggressive motion and degraded localization, the disturbance shape is defined in Equation (18) as

(18) $$S_{k}=diag\left(\sigma_{p,k}^{2},\sigma_{p,k}^{2},\sigma_{\theta ,k}^{2}\right),\;\;\sigma_{p,k}=\sigma_{p,0}+c_{v}\left|{\bar{v}}_{k}\right|+c_{\omega }\left|{\bar{\omega }}_{k}\right|+c_{l}tr\left(\Sigma_{loc,k}\right),\sigma_{\theta ,k}=\sigma_{\theta ,0}+c_{\theta }\left|{\bar{\omega }}_{k}\right|,$$

where ${\bar{v}}_{k}\;$and ${\bar{\omega }}_{k}$ are the nominal translational and angular inputs, $\Sigma_{loc,k}\;$is the pose-localization covariance, and $c_{v}$, $c_{\omega }$, $c_{l}$, and $c_{\theta }$ are nonnegative tuning coefficients.

Equation (18) shows that the disturbance envelope expands automatically when the relay moves faster, turns more sharply, or becomes less certain about its own pose. Based on the closed-loop error dynamics in Equation (16), the exact tube propagation is written in Equation (19) as

(19) $$E_{k+1}⊇(A_{k}+B_{k}K_{k})E_{k}⊕W_{k},$$

where ⊕ denotes the Minkowski sum. Because the exact set propagation in Equation (19) is too expensive to compute online, a conservative scalar recursion is introduced. The induced radius contribution of the disturbance set under the P-metric is defined in Equation (20) as

(20) $$d_{k}=\sqrt{\lambda_{max}\left(P^{-1/2}S_{k}P^{-1/2}\right)},$$

and let $\lambda_{k}\in (0,1)$ denote a contraction coefficient satisfying $\left(A_{k}+B_{k}K_{k})E_{k}\subseteq \{e:e^{⊤}P^{-1}e\leq \lambda_{k}^{2}\rho_{k}^{2}\right\}$. Then, a sufficient scalar radius recursion is obtained in Equation (21) as

(21) $$\rho_{k+1}=\lambda_{k}\rho_{k}+d_{k}.$$

Equation (21) captures the competition between feedback contraction and disturbance growth. If the initial error satisfies ${\tilde{e}}_{0}\in E_{0}$ and the recursion in Equation (21) holds along the prediction horizon, then the actual tracking error remains inside the propagated tube.

To translate the tube radius into a bound on the planar relay position, let the position-extraction matrix be defined as $C_{z}=[I_{2}\;\;\;\;0].$ Using the tube definition in Equation (17), the induced planar deviation bound is written in Equation (22) as

(22) $$dist(z_{k},{\bar{z}}_{k})\leq b_{k}=\rho_{k}\sqrt{\lambda_{max}(C_{z}PC_{z}^{⊤})},$$

where ${\bar{z}}_{k}=C_{z}{\hat{s}}_{k}$ denotes the nominal planar position. Equation (22) therefore provides a compact bound on the maximum planar deviation between the actual relay position and the nominal trajectory at step k.

### 4.3. Tube-Tightened Communication and Safety Constraints

Since the actual planar position lies inside the tube-centered neighborhood $B({\bar{z}}_{k},b_{k})$, the local communication variation bound derived in Equation (12) can now be combined with the tube geometry. Specifically, the smoothness argument implies that the worst-case conservative communication level over the tube satisfies $LCB(z_{k})\geq LCB({\bar{z}}_{k})-{\hat{L}}_{k}b_{k}$.

Based on this relation, a sufficient model-based condition for communication preservation over the full closed-loop tube is written in Equation (23) as

(23) $$LCB({\bar{z}}_{k})-{\hat{L}}_{k}b_{k}\geq r_{min}-\xi_{k},\xi_{k}\geq 0,$$

where $r_{min}$ is the operational RSSI threshold and $\xi_{k}$ is a nonnegative communication slack variable. Using the lower confidence bound defined earlier in Equation (10), Equation (23) can be rewritten equivalently as Equation (24):

(24) $$\mu_{r}({\bar{z}}_{k})-\beta \sigma_{r}({\bar{z}}_{k})-{\hat{L}}_{k}b_{k}\geq r_{min}-\xi_{k}.$$

Equation (24) is the key robustification step of the proposed controller. The term $\mu_{r}({\bar{z}}_{k})-\beta \sigma_{r}({\bar{z}}_{k})$ accounts for radio-prediction uncertainty through the conservative communication field, whereas the additional term ${\hat{L}}_{k}b_{k}$ accounts for motion-tracking uncertainty through the tube-induced positional deviation. As a result, communication preservation is enforced over the propagated tube rather than only at the nominal predicted state.

To preserve recursive feasibility when communication degradation is temporarily unavoidable, the slack variable $\xi_{k}$ is included and heavily penalized in the MPC cost. In this way, temporary threshold violation is allowed only when strictly necessary.

The tube is also used to enforce collision avoidance conservatively. Let the obstacle-free workspace be represented by local safety functions $h_{i}(z)\geq 0$, $i=1,\ldots ,M_{o}$, which are positive in the safe region and vanish on obstacle boundaries. Linearizing the safety function around the nominal position and requiring safety for all actual positions inside the tube gives the tightened obstacle condition in Equation (25):

(25) $$h_{i}({\bar{z}}_{k})-∥\nabla h_{i}({\bar{z}}_{k}){∥}_{2}\;b_{k}\geq d_{body},i=1,\ldots ,M_{o},$$

where $d_{body}$ is the nominal body-clearance margin. Because the MPC optimizes only the nominal control input, the admissible input and state sets must also be tightened consistently with the deviation tube. The resulting tightened constraints are written in Equation (26) as

(26) $${\bar{u}}_{k}\in U⊖K_{k}E_{k},{\hat{s}}_{k}\in X⊖E_{k},$$

where ⊖ denotes the Pontryagin difference and X is the admissible nominal state set. Taken together, Equations (25) and (26) ensure that obstacle avoidance and actuator limits are satisfied for all actual trajectories consistent with the propagated tube.

### 4.4. Finite-Horizon Optimization Problem

Collecting the nominal dynamics, the tube propagation rule, and the tightened communication and safety constraints, the finite-horizon optimal control problem at time step k is formulated in Equation (27) as

(27) $$\begin{array}{l} \underset{\left\{{\bar{u}}_{j|k},\rho_{j|k},\xi_{j|k}\right\}}{\min}\sum_{j=0}^{N_{p}-1}\left({\left\|C_{z}{\hat{s}}_{j|k}-z_{j|k}^{ref}\right\|}_{Q}^{2}+{\left\|{\bar{u}}_{j|k}\right\|}_{R}^{2}+\alpha \rho_{j|k}^{2}+\gamma \xi_{j|k}^{2}\right)\\ \;\;\;\;\;\;+{\left\|C_{z}{\hat{s}}_{j|k}-z_{j|k}^{ref}\right\|}_{Q_{f}}^{2}\\ \;\;s.\;t.\;\;{\hat{s}}_{0|k}=s_{k},\;\;\rho_{0|k}=\rho_{k},\\ \;\;\;\;{\hat{s}}_{j+1|k}=A_{j|k}{\hat{s}}_{j|k}+B_{j|k}{\bar{u}}_{j|k}+c_{j|k},\\ \;\;\;\;\mu_{r}\left({\bar{z}}_{j|k}\right)-\beta \sigma_{r}\left({\bar{z}}_{j|k}\right)-{\hat{L}}_{j|k}b_{j|k}\geq r_{min}-\xi_{j|k},\\ \;\;\;\;h_{i}\left({\bar{z}}_{j|k}\right)-{\left\|\nabla h_{i}\left({\bar{z}}_{j|k}\right)\right\|}_{2}b_{j|k}\geq d_{body},\\ \;\;\;\;{\bar{u}}_{j|k}\in U⊖K_{j|k}E_{j|k},\\ \;\;\;\;{\hat{s}}_{j|k}\in X⊖E_{j|k},\;\;\xi_{j|k}\geq 0, \end{array}$$

where the dynamics, tube, communication, obstacle, and input constraints hold for $j=0,\ldots ,N_{p}-1$ and $i=1,\ldots ,M_{o},$ and the state inclusion ${\hat{s}}_{j|k}\in X⊖E_{j|k}$ holds for $j=0,\ldots ,N_{p}.$ In Equation (27), the first term promotes nominal reference tracking, the second penalizes control effort, the third discourages excessive tube growth, and the fourth penalizes communication-threshold violation through the slack variable. The terminal penalty further promotes convergence toward the desired support position over the prediction horizon.

For completeness, the optimization in Equation (27) is solved subject to the current nominal initial condition ${\hat{s}}_{0}=s_{k}$, while $\rho_{0}$ is initialized from the current tracking-error estimate or from a prescribed tube-initialization rule. Only the first optimal nominal control action ${\bar{u}}_{k}^{*}$ is then applied through $u_{k}={\bar{u}}_{k}^{*}+K_{k}{\tilde{e}}_{k}$, and the optimization is repeated at the next sampling instant using updated state and radio measurements.

The proposed controller therefore provides robustness at two coupled levels. The GPR-based radio-perception layer penalizes spatially uncertain regions through Equation (10), discouraging entry into poorly observed NLOS regions, while the adaptive tube tightens both communication and obstacle constraints through Equations (24)–(26) using a dynamically evolving uncertainty margin rather than a fixed buffer.

### 4.5. Recursive Feasibility and Stability

The tube formulation admits the standard guarantees of robust tube MPC, namely tube invariance, recursive feasibility, and uniform ultimate boundedness (UUB) of the tracking error. The only nonstandard element is the non-holonomic transition $A_{k}+B_{k}K_{k}$, which depends on the nominal heading through $A_{k}+B_{k}K_{k}=T_{k+1}\;F_{b}T_{k}^{⊤}$ with $T_{k}=blkdiag(R({\bar{\theta }}_{k}),1)$, so a single constant matrix cannot certify a uniform contraction. The shape matrix P of Equation (17) is therefore taken in the heading-aligned frame, $P_{k}=T_{k}\;P_{b}T_{k}^{⊤}$, where the constant body-frame matrix $P_{b}$ and a single rate λ∈(0,1) are obtained once offline from the contraction LMI

(28) $$F_{b}(\bar{v},\bar{\omega }{)}^{⊤}P_{b}^{-1}F_{b}(\bar{v},\bar{\omega })-\lambda^{2}P_{b}^{-1}⪯0,\forall (\bar{v},\bar{\omega })\in 𝒱,$$

where $F_{b}(\bar{v},\bar{\omega })=T_{k+1}^{⊤}(A_{k}+B_{k}K_{k})T_{k}$ is heading-independent and 𝒱 is the compact velocity envelope. Because the position component of the disturbance is isotropic, $\lambda_{max}(C_{z}P_{b}C_{z}^{⊤})$ is heading-invariant, so the disturbance radius in Equation (20) and the planar bound in Equation (22) are numerically unchanged, and the per-step coefficient in Equation (21) satisfies $\lambda_{k}\leq \lambda <1$ with a certificate uniform over the operating range.

**Proposition 1**  (**Tube invariance**)**.** *With *$P_{k}$* from Equation (28) and *$\rho_{k}$* propagated by Equation (21), *$e_{k}\in E_{k}$* implies *$e_{k+1}\in E_{k+1}$* for all *$w_{k}\in W_{k}$*. In body coordinates *$\eta_{k}=T_{k}^{⊤}e_{k}$* the error obeys *$\eta_{k+1}=F_{b}\eta_{k}+\nu_{k}$*; Equation (28) gives *$∥F_{b}\eta_{k}{∥}_{P_{b}^{-1}}\leq \lambda_{k}\rho_{k}$*, and isotropy gives *$∥\nu_{k}{∥}_{P_{b}^{-1}}\leq d_{k}$*, so *$∥\eta_{k+1}{∥}_{P_{b}^{-1}}\leq \lambda_{k}\rho_{k}+d_{k}=\rho_{k+1}$*. Iterating with *λ<1* bounds the radius by *$\rho_{\infty }=\frac{d_{max}}{1-\lambda }$*, so the tube, and hence bk in Equation (22), is uniformly bounded over arbitrarily long missions. This is what allows the tightened constraints (23)–(26) to be enforced over the tube rather than only at the nominal state.*

**Proposition 2**  (**Recursive feasibility**)**.** *Under bounded disturbance, nonempty tightened hard-constraint sets, a feasible terminal set, and sufficiently large nonnegative communication slack, the feasibility of the softened MPC problem is recursively preserved. A feasible candidate at k + 1 is the shifted optimal step-k plan completed by the terminal feedback *${\bar{u}}_{N}=K_{k}({\bar{s}}_{N}-{\bar{s}}_{ref})$* on the heading-independent terminal set *$X_{f}=\left\{\;s\in X⊖{\bar{E}}_{\infty }:∥C_{z}s-z^{\mathrm{ref}}{∥}_{2}\leq b_{\infty }\;\right\}$*. The one-step relinearization and radio-prediction mismatch are absorbed by the bounded disturbance and the nonnegative slack* $\xi_{k}$*; the Pontryagin-tightened sets in Equation (26) stay nonempty because the radii are uniformly bounded by Proposition 1 and* $0\in U⊖K_{k}E_{k}$* by design. A hold-position terminal action is excluded because the unicycle nominal transition has all eigenvalues equal to one, so the stabilizing feedback is required.*

**Proposition 3**  (**Practical stability, UUB**)**.** *Suppose *$z_{k}^{\mathrm{ref}}$ *is constant over a sufficient interval, and the terminal weight *$Q_{f}$* satisfies the terminal Lyapunov condition *${F_{b}}^{⊤}Q_{f,b}F_{b}+Q+{K_{b}}^{⊤}R\;K_{b}⪯Q_{f,b}$* over *𝒱*, with *$Q_{f,b}$* the discrete Riccati matrix for (*$F_{b},\;Q,\;R$*). Then the MPC value function of Equation (27) is a Lyapunov function whose one-step decrease is a class-𝒦 function of the tracking error minus a disturbance-dependent term, so the nominal error is UUB and, by the tube containment of Proposition 1, the actual planar error satisfies*

(29) $$∥\;C_{z}s_{k}-z^{\mathrm{ref}}{∥}_{2}\;\;\leq \;\;b_{\mathrm{nom}}+b_{\infty }+\epsilon ,\forall k\geq T,$$

*for every* ϵ>0 *and a finite settling time* T *. The closed loop therefore converges to a neighborhood of radius* $b_{\mathrm{nom}}+b_{\infty }$ *set by the residual radio-prediction and motion uncertainty rather than to the reference itself, which is practical stability under the persistent bounded disturbance. Taken together, Propositions 1–3 certify that the tightened constraints are enforced over a provably invariant tube, that the softened MPC problem is recursively feasible under the stated assumptions, and that the tracking error is confined to a bounded neighborhood governed by the uncertainty the controller rejects.*

## 5. Simulation Results

This section evaluates the proposed Gaussian Process-Driven Adaptive Tube Model Predictive Control (GP-ATMPC) in two representative indoor disaster environments. The objective of the simulation study is to examine whether the proposed controller can preserve communication more reliably than the compared baselines while simultaneously maintaining collision-free relay motion and sufficient exploration progress. To this end, the simulation environment is first described. A component-wise ablation illustration follows, and the main 50-run Monte Carlo comparison is then presented. RSSI tracking behavior and time-domain communication performance are subsequently analyzed, and the spatial relay deployment is finally interpreted.

### 5.1. Simulation Setup

We evaluate the proposed framework in two indoor disaster environments of increasing difficulty: a Three-Room environment with corridor and doorway bottlenecks, and a larger Warehouse environment with longer corridors and additional non-line-of-sight (NLOS) rooms. Using two environments rather than one tests whether the conclusions hold as the scenario scales in size and connectivity stress. In each environment, an explorer follows a fixed reference path from a base station while a chain of mobile relays is deployed on demand to preserve an end-to-end communication link. The relay count is sized automatically from the base-to-goal route and the maximum hop distance of 12 m, giving about four relays in the Three-Room environment and up to eight in the Warehouse environment.

Four controllers are compared under a common chain-balancing scheduler: the proposed GP-ATMPC, nominal MPC, heuristic adaptive-margin MPC, and Reactive Connectivity-Aware Relay Algorithm (CARA) [45]. To ensure a fair comparison, the model predictive methods use the same local quadratic-programming MPC structure, ancillary feedback law, tube propagation, obstacle tightening, input tightening, and sequential relay-planning scheme. The scheduler supplies the reference support position $z_{i,k}^{\mathrm{ref}}$ for each relay i at step k as defined in Equation (30),

(30) $$z_{i,k}^{\mathrm{ref}}=arg\;\underset{z\in \Omega_{i,k}}{max}\;min\{C(z,p_{\pi_{i},k}),C(z,p_{\chi_{i},k})\},\;$$

where $\pi_{i}$ and $\chi_{i}$ denote the upstream parent and downstream child of relay i, $C(z,p)=r_{\mathrm{nom}}(d(z,p))$ is the nominal-path-loss score, and $\Omega_{i,k}$ is a local candidate set generated within a 1.5 m search radius around a parent–child interpolated seed and projected into the obstacle-free workspace. The downstream child of the last relay is the explorer.

All controllers share the motion model, tube propagation, obstacle tightening, and input tightening; they differ only in the communication-safety term $P_{j|k}^{\mathrm{safe}}$ enforced by $P_{j|k}^{\mathrm{safe}}\geq r_{min}-\xi_{j|k}$. The proposed GP-ATMPC uses Equation (24). Nominal MPC uses $r_{\mathrm{nom}}\left(d\left({\bar{z}}_{j|k},p_{j|k}\right)\right)\;$with no margin. Heuristic adaptive-margin MPC subtracts a hand-designed buffer $M_{j|k}=M_{0}+M_{v}|{\bar{v}}_{j|k}|+\;M_{\omega }|{\bar{\omega }}_{j|k}|+\;M_{\Sigma }\sqrt{\mathrm{tr}(\Sigma_{\mathrm{loc},j|k}^{xy})}$ with $\left(M_{0},M_{v},M_{\omega },M_{\Sigma })=(5,10,5,8\right)$, where ${\bar{v}}_{j|k}$ and ${\bar{\omega }}_{j|k}$ are the nominal linear and angular speeds and $M_{\Sigma }\sqrt{\mathrm{tr}(\Sigma_{\mathrm{loc},j|k}^{xy})}$ is the position block of the localization covariance. Reactive CARA does not use the receding-horizon formulation; it repositions each relay toward the weaker neighbor whenever the worst measured link approaches the threshold, and therefore represents a connectivity-first design from a different family.

The radio channel uses a log-distance model fitted to real corridor measurements, with received power $r_{0}=-13.3$ dBm at 1 m and path-loss exponent n=2.42, so that the simulated and measured channels are consistent. A boolean NLOS excess loss of 9 dB is applied whenever the relay-parent line segment intersects an obstacle. The ground-truth shadowing residual used to generate measurements, and not directly available to the controller, consists of Gaussian attenuation bumps with amplitudes between 5 and 9 dB and length scales of 0.8 to 1.0 m placed near corridor and doorway midpoints. Measurement noise is added with a standard deviation $\sigma_{\varepsilon }$ = 1.5 dB, and the connectivity threshold is$\;r_{min}=-65\;dBm$. The GP layer learns this residual field online from RSSI observations as described in Section 3.

We report results over 50 Monte Carlo runs per environment. The primary connectivity metric is the satisfaction rate (Sat), the percentage of mission steps whose end-to-end worst-link RSSI is at or above $r_{min}$, reported together with the end-to-end violation count and the average and minimum worst-link RSSI. Physical safety is reported through the near-collision rate, the percentage of steps in which a relay is rescued from a wall or obstacle by the safety projection. We use Sat and the safety metrics rather than a binary mission-success count as the primary criterion, because binary success depends on an absolute per-step threshold that is sensitive to scenario length and, by construction, credits unsafe wall-proximity behavior; for completeness, the mission-success thresholds of $V_{max}^{tot}=150\;$ total and $V_{max}^{cons}=20$ consecutive below-threshold steps are retained for the reference success figures. The key simulation, controller, and learning parameters are summarized in Table 1. All analytical Monte Carlo simulations were implemented in MATLAB R2025b.

### 5.2. Component-Wise Ablation Study

**Figure 1:**
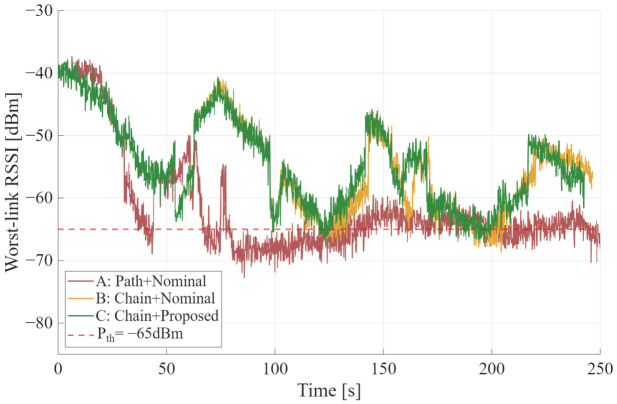
Ablation: end-to-end worst-link RSSI over time.

To separate the roles of the chain-balancing scheduler and the communication-aware controller, we run a component-wise ablation in the Three-Room environment over 50 runs. Configuration A pairs a path-based scheduler with the nominal controller, B pairs the chain-balancing scheduler with the nominal controller, and C is the full proposed framework. Table 2 and Figure 1 summarize the results.

The path-based baseline A satisfies the connectivity constraint at only 94.67% with 264 ± 64 violations, because the relays follow geometrically convenient but communication-unfavorable positions. Replacing only the scheduler (A to B) cuts violations to 48 ± 20, about 5.5 times fewer, and raises Sat to 99.01%, confirming that relay placement dominates the outcome. Adding the proposed controller on top (B to C) further reduces violations to 38 ± 15, about 20% fewer, edges Sat to 99.21%, and tightens the standard deviation from 20 to 15. The scheduler is primarily responsible for enabling connectivity-aware progress, and the uncertainty-aware controller supplies the residual, more consistent margin. Figure 1 shows the same effect in the time domain: configuration A spends long intervals below the threshold, while B and C stay above it for nearly the entire run.

### 5.3. Three-Room Environment

Table 3 reports the Three-Room environment over 50 Monte Carlo runs. All four controllers complete the mission with full explorer progress in this topology, so the comparison turns on how reliably the connectivity constraint is satisfied and at what safety cost. Among the principled controllers, the proposed GP-ATMPC attains the highest Sat at 99.21%, with the lowest violation count at 38 ± 15, about 20% fewer than nominal MPC (48 ± 20) and 22% fewer than the heuristic adaptive-margin baseline (49 ± 25), and the strongest minimum worst-link RSSI at −68.1 dBm. A paired Wilcoxon signed-rank test confirms that the reductions in violation count relative to nominal and heuristic MPC are significant ($p\;=\;8.0\;\times \;10^{-4}\;and\;4.6\;\times \;10^{-3}$).

Reactive CARA reaches a marginally higher Sat of 99.79% with only 10 ± 3 violations, the best raw connectivity of the four methods. This advantage is not free: its near-collision rate is 16.6% (1715 events) against 4.5% (460) for the proposed method, about 3.7 times higher, because CARA preserves the link by driving relays aggressively close to walls and doorways. The proposed method therefore achieves comparable, near-saturated connectivity in this easy topology at roughly one-third of the unsafe-proximity events, and unlike CARA, it carries the recursive feasibility and stability guarantees of Section 4.5. All model-predictive controllers solve in about 5 ms per step, well within real-time, while CARA’s reactive rule is cheaper at about 0.1 ms but provides none of the predictive constraint handling.

**Figure 2:**
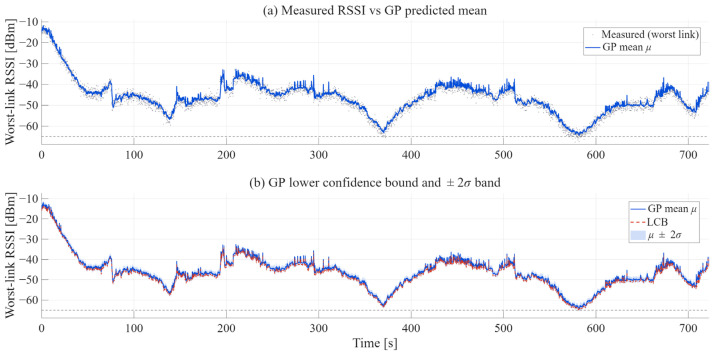
RSSI tracking and predictive uncertainty for the proposed method (Three-Room).

Figure 2 examines whether the radio layer captures the measured channel meaningfully, using the proposed method’s representative run. Figure 2a compares the measured end-to-end worst-link RSSI with the pre-update GP predicted mean, so it reflects genuine prediction rather than posterior reconstruction; the mean follows the channel trend, including the deep attenuation near the far rooms. Figure 2b shows the GP mean with its lower confidence bound (LCB) and ±2σ band: the band widens in sparsely observed regions, and the LCB drops ahead of the measured RSSI at doorways and partially shadowed transitions, acting as a conservative early-warning signal that the controller reacts to.

**Figure 3:**
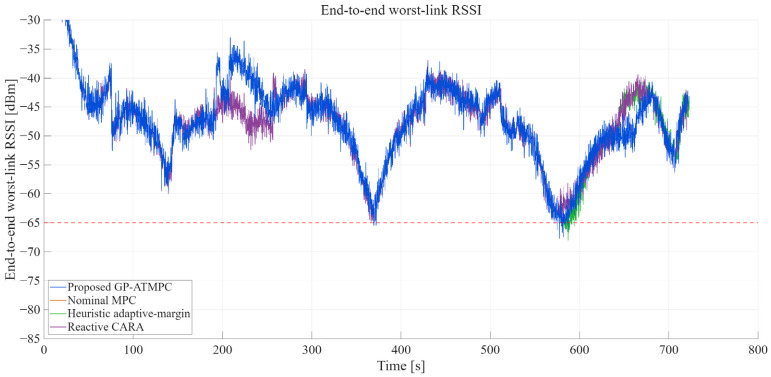
End-to-end worst-link RSSI over the mission horizon (Three-Room).

Figure 3 shows the end-to-end worst-link RSSI over the mission for the four methods in a representative run. The worst-link RSSI stays well above the −65 dBm threshold for most of the horizon and is stressed only in the far region, where the explorer is deepest in the environment and the chain is fully extended. There, the principled controllers show brief, shallow excursions toward −67 to −68 dBm, while CARA holds marginally above the threshold throughout; the aggregate of these excursions across all 50 runs is the violation and Sat statistics in Table 3. The figure thus localizes where connectivity is hardest to maintain.

**Figure 4:**
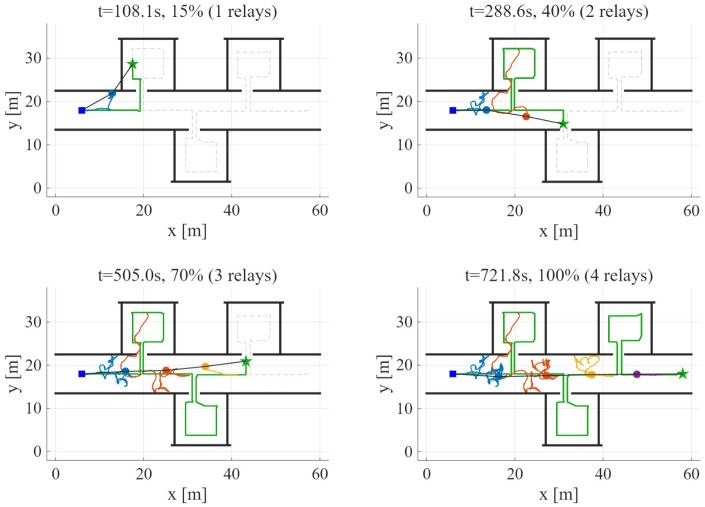
Relay-chain spatial redistribution for the proposed method (Three-Room).

Figure 4 shows representative snapshots of the proposed relay chain. In each snapshot, the base station is marked by a square, the explorer by a star, and the deployed relays by colored markers with their recent trajectories; the subplot titles give the mission time, explorer progress, and active relay count. Early in the run, the relays remain concentrated near the base while the explorer is still close. As the explorer crosses corridor transitions and doorways, the relays redistribute into intermediate support positions rather than holding fixed spacing, and by the late stage, the chain spans the explored area while keeping a structured, communication-aware formation. This redistribution is the spatial explanation for the maintained worst-link RSSI and reduced violation counts.

### 5.4. Warehouse Environment

Table 4 reports the larger Warehouse environment over 50 runs, where longer corridors and additional NLOS rooms make connectivity preservation substantially harder. Here the proposed GP-ATMPC clearly separates from the same-family baselines: it raises Sat from 92.74% (nominal) and 92.60% (heuristic) to 96.56%, improves the average and minimum worst-link RSSI to −48.7 and −69.1 dBm, and is the only controller in this family that drives the explorer fully to the goal, at 100% progress versus 98.2% with the baselines stalling about 3.5 m short. Near-collision rates stay at the baseline level, 5.7% versus 5.5 to 5.6%, so the connectivity gain does not cost safety relative to the other principled controllers.

The violation-count means in this environment are inflated by a small number of difficult seeds, as reflected in the large standard deviations. The median violation count of the proposed method is 52, against 118 and 130 for nominal and heuristic MPC, and a paired Wilcoxon signed-rank test confirms that the proposed method significantly reduces violations and increases Sat relative to both same-family baselines (*p* < 10^−4^ in all cases). The 95% confidence interval for the proposed method’s Sat is 94.6 to 98.5%.

Reactive CARA again attains the best raw connectivity, Sat 99.97% with 2 ± 1 violations, but at a 20.0% near-collision rate (4691 events), about 3.5 times the proposed method’s 5.7% (1379), reflecting wall-proximity link preservation with no feasibility or stability guarantee. For reference, binary mission success in this environment was 78% for the proposed method, 52% for nominal, 50% for heuristic, and 100% for CARA; this metric credits CARA’s unsafe behavior and is sensitive to scenario length, which is why we report Sat and the safety metrics as the primary criteria.

**Figure 5:**
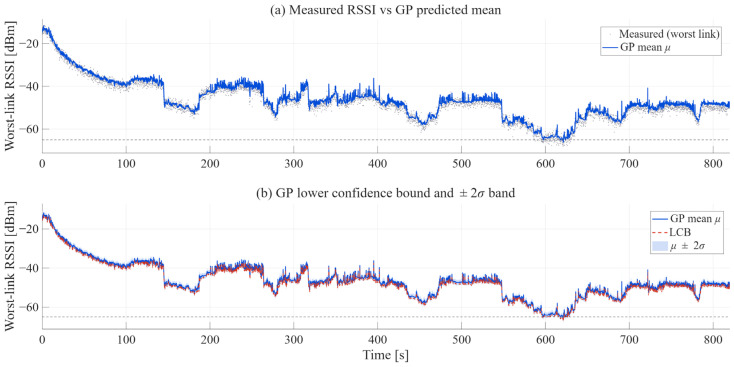
RSSI tracking and predictive uncertainty for the proposed method (Warehouse).

Figure 5 repeats the radio-layer check for the Warehouse environment using the proposed method’s representative run, in the same format as Figure 2. The longer corridors and additional NLOS rooms produce deeper and more frequent attenuation events, and the uncertainty band widens more strongly at the far-room transitions, where the chain has extended to eight relays. The LCB again drops ahead of the measured RSSI at these transitions.

**Figure 6:**
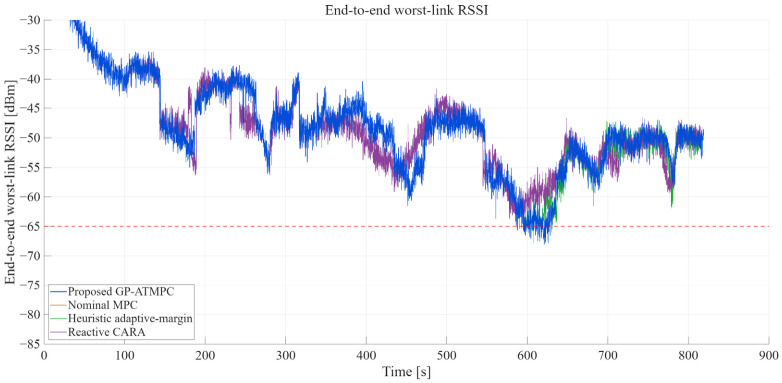
End-to-end worst-link RSSI over the mission horizon (Warehouse).

Figure 6 shows the end-to-end worst-link RSSI over the mission for the four methods in a representative Warehouse run, in the same format as Figure 3. Connectivity is comfortably maintained through the first part of the route and becomes stressed only after about 590 s, when the explorer reaches the far rooms and the chain is fully extended. The excursions there are deeper and more frequent than in the Three-Room environment, consistent with the lower Sat in Table 4; CARA holds marginally above the threshold throughout. The run-to-run comparison across all 50 seeds is summarized in Table 4.

**Figure 7:**
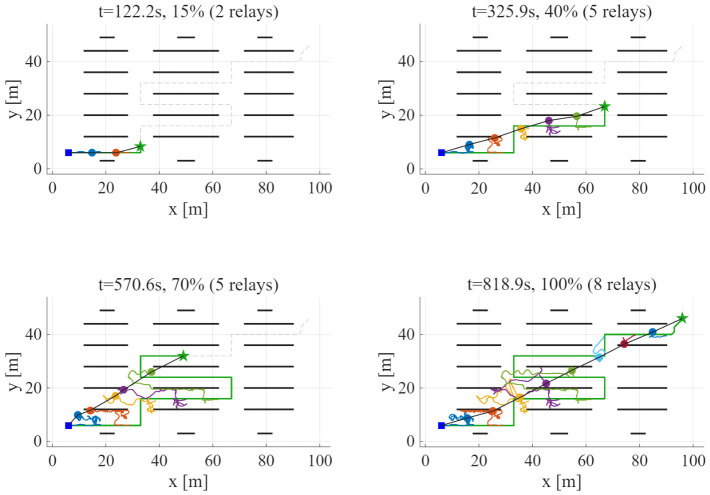
Relay-chain spatial redistribution for the proposed method (Warehouse).

Figure 7 shows representative snapshots of the proposed relay chain in the Warehouse environment, in the same format as Figure 4. The chain grows to eight relays and spans the full multi-room layout, redistributing through successive doorways as the explorer advances. The larger span and relay count are the spatial counterparts of the harder connectivity-preservation task reflected in the Warehouse statistics.

**Figure 8:**
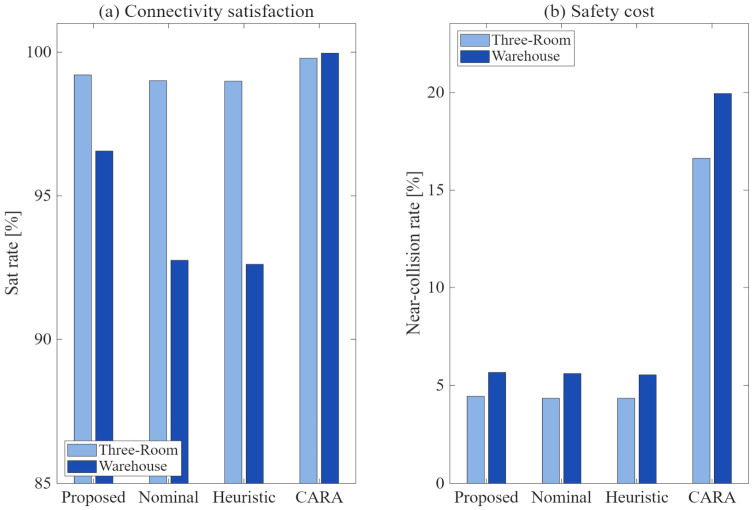
Connectivity and safety trade-off across both environments.

Figure 8 summarizes the trade-off across both environments. Figure 8a plots the Sat and Figure 8b the near-collision rate for the four methods. The two panels make the central trade-off explicit: Reactive CARA attains the highest Sat in both environments but also the highest near-collision rate, about 3.5 to 3.7 times that of the proposed method, whereas the proposed method attains the highest Sat among the controllers that preserve a safe margin, at near-baseline near-collision rates. Plotting connectivity and safety together prevents the unsafe wall-proximity behavior from being read as a genuine performance advantage.

### 5.5. Simulation Validation

**Figure 9:**
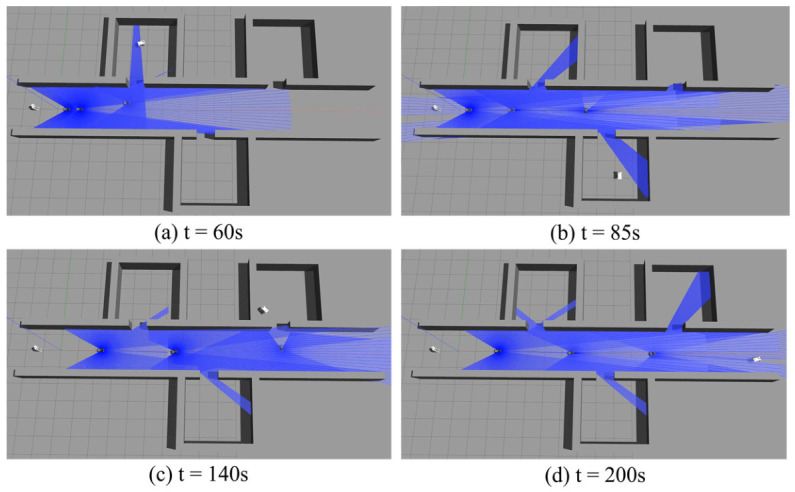
Time-ordered snapshots of a representative run in the Gazebo multi-room environment.

**Figure 10:**
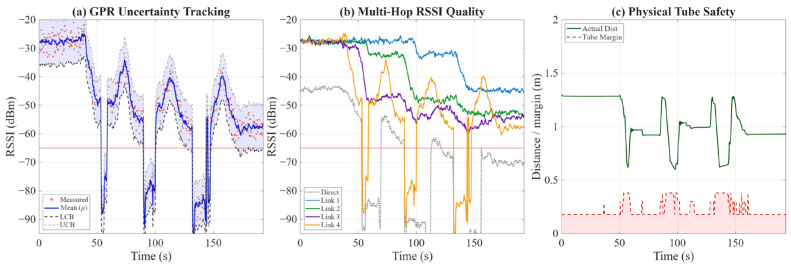
Gazebo validation of the proposed method over a representative multi-room run.

To bridge the analytical simulations of Section 5.3 and Section 5.4 and a physical deployment, the proposed method was evaluated in a Gazebo environment (built on ROS 2 Humble) in which the radio field, robot dynamics, and sensing are subject to higher-fidelity disturbances than the analytical channel model. Figure 9 and Figure 10 summarize a representative run through a multi-room layout in which the explorer repeatedly enters non-line-of-sight (NLOS) rooms while the relay chain follows: Figure 9 shows the spatial scene and Figure 10 the time-domain signals.

Figure 9 shows four time-ordered snapshots of the run viewed from above. The blue fans are the onboard laser scans of the explorer and the relays, so each robot’s footprint marks its locally sensed free space. In the first snapshot, the explorer (white) and the relay chain are still grouped near the base region on the left. As the run proceeds, the explorer advances into successive rooms, including a non-line-of-sight room off the main corridor, while the relays do not simply trail at fixed spacing but redistribute toward the doorways and corridor junctions that carry the active communication path. By the final snapshot, the chain spans the full width of the explored area, with relays held at the corridor openings so that the explorer retains an end-to-end link even after entering the far rooms. This spatial behavior is the higher-fidelity counterpart of the relay redistribution reported in Section 5.3, and it sets the scene for the time-domain signals examined next.

Figure 10a shows the GP radio-prediction layer operating online. The posterior mean tracks the measured RSSI across the run, and the lower confidence bound (LCB) tracks below the mean and lower-bounds the bulk of the measurements, including the sharp NLOS drops near 50, 95, and 130 s where the signal falls from roughly −28 dBm to below −85 dBm. The uncertainty band widens at these transitions, so the LCB stays conservative exactly where the channel is least predictable, which is the property exploited by the connectivity constraint in Section 3.3. Figure 10b contrasts the direct explorer-to-base link with the four relayed hops. The direct link degrades past the −65 dBm connectivity threshold after about 50 s and falls toward −90 dBm inside the rooms, whereas the relayed links remain near or above the threshold for the duration of the run; the most recently deployed frontier hop (Link 4) is the noisiest, as expected for the actively repositioning relay. Figure 10c reports the physical tube safety. The realized inter-robot clearance (Actual Dist) stays above the tube margin throughout, never entering the shaded tube-violation region even during the aggressive maneuvers where the clearance briefly drops to about 0.6 m against a margin of about 0.2 to 0.4 m. This is the empirical counterpart of the recursive feasibility and constraint-satisfaction guarantees established in Section 4.5.

These results indicate that the radio prediction, multi-hop relaying, and tube-based safety mechanisms continue to function under the richer disturbances of the Gazebo environment. We emphasize that this remains a higher-fidelity simulation study, not a hardware deployment; a full physical multi-robot experiment with live networking is left as future work.

### 5.6. Validation on Real Indoor RSSI Measurements

To verify that the GP radio-prediction layer remains effective under real channel fluctuations rather than only on analytically generated fields, the GP was evaluated on RSSI measurements collected on the 4th floor of a campus building. A mobile robot traversed a corridor and a non-line-of-sight (NLOS) room at a constant speed of 0.06 m/s, so that the measurement timestamps map directly to a one-dimensional path coordinate. The GP was trained on sparsely sampled measurements (about one sample per 0.5 m) and used to predict the RSSI at the remaining unobserved locations.

**Figure 11:**
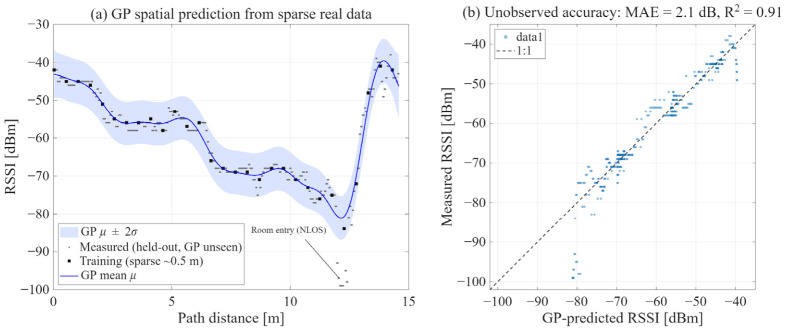
GP validation on real indoor RSSI measurements.

As shown in Figure 11a, the GP mean reconstructs the measured spatial RSSI profile, including the sharp attenuation on room entry, while the posterior uncertainty band widens in sparsely observed regions. On the held-out locations, the prediction attains a mean absolute error of about 2.1 dB with R^2^ ≈ 0.91 for the NLOS trajectory and about 4.9 dB with R^2^ ≈ 0.85 for a longer corridor run. The parity plot in Figure 11b shows the held-out predictions clustering along the 1:1 line, with the largest deviations confined to the deep NLOS dip below −80 dBm. The fitted length scale of about 0.9 to 1.5 m is consistent with the kernel setting used throughout the paper. The GP here is applied over the traversed path coordinate; the spatial-prediction and uncertainty-calibration behavior is the same as that exploited by the lower confidence bound in Section 3.3. These results confirm that the GP predicts real indoor RSSI with low error and reports growing uncertainty exactly where measurements are sparse.

## 6. Conclusions

This paper proposes a Gaussian Process-Driven Adaptive Tube Model Predictive Control (GP-ATMPC) framework for communication-preserving navigation of mobile relay robots in indoor disaster environments. The proposed method combines residual-based Gaussian process regression (GPR) radio prediction with adaptive tube MPC so that communication preservation is enforced through a conservative tube-tightened condition under coupled radio and motion uncertainty.

Simulation results across two indoor disaster environments and 50 Monte Carlo runs each showed that, among the controllers that preserve a safe motion margin, the proposed GP-ATMPC achieved the highest connectivity satisfaction rate and the strongest average and minimum end-to-end worst-link RSSI, with significantly fewer connectivity violations than nominal and heuristic adaptive-margin MPC, while maintaining millisecond-level online solve times. A reactive connectivity-first baseline reached higher raw connectivity but at three to four times the near-collision rate and without feasibility or stability guarantees, so the proposed method provides the most favorable balance among communication continuity, collision-free motion, and forward mission execution among the principled controllers. Beyond the analytical simulations, the radio prediction and tube-based safety behavior of the proposed method were further examined in a Gazebo environment and on real indoor RSSI measurements collected on a building floor, where the Gaussian process reconstructed the measured channel with a mean absolute error of about 2.1 dB and reported growing uncertainty in sparsely observed regions.

The proposed framework should be interpreted not as a hard empirical guarantee of threshold satisfaction at every time step, but as a practical and robust communication-preserving navigation strategy. The present validation spans analytical simulation, a higher-fidelity Gazebo environment, and real indoor RSSI measurements; a full physical multi-robot deployment with live networking has not yet been performed. Future work will therefore pursue such a deployment, together with cooperative multi-relay coordination and larger-scale Gaussian process approximations.

## Figures and Tables

**Table 1:** Core simulation parameters.

Method	Parameter	Value
Time/horizon	$T_{s}/N_{p}$	0.15 s/8
Robot limits	$v_{max}/\omega_{max}/d_{body}$	0.75 m/s/1.4 rad/s/0.22 m
Communication	$r_{min}/\beta /\kappa$	−65 dBm/1.2/1.2
Radio model	$r_{0}$, n, NLOS loss	−13.3 dBm @ 1 m, 2.42, 9 dB
MPC weights	$Q/R/Q_{f}$	diag(5, 5)/diag(1.5, 0.8)/diag(10, 10)
Tube weights	$\alpha /\gamma /P/\rho_{0}/\rho_{max}$	10/30/diag(0.04, 0.04, 0.02)/0.02/1.5
Disturbance	$\left(\sigma_{p,0},\sigma_{\theta ,0}\right)$, $\left(c_{v},c_{\omega },c_{l},c_{\theta }\right)$	(0.008 m, 0.014 rad), (0.018, 0.010, 0.10, 0.020)
GP kernel	$\sigma_{f}/l/\sigma_{n}$	5 dB/1.0–1.5 m/1.5 dB
Mission success	$V_{max}^{tot}/V_{max}^{cons}$	150/20 steps
Baselines	$\left(M_{0},M_{v},M_{\omega },M_{\Sigma }\right)$	(5, 10, 5, 8)
Reactive CARA	hysteresis, blend, $k_{v},\;k_{\omega }$	3 dB, 0.6, 0.9, 1.6

**Table 2:** Component-wise ablation.

Method	Sat [%]	E2E Viol.	E2E Avg. RSSI [dBm]	E2E min RSSI [dBm]	Near-Coll. [%] (Count)
A: Path + Nominal	94.67	264 ± 64	−54.2	−70.1	0.3 (36)
B: Chain + Nominal	99.01	48 ± 20	−47.8	−68.4	4.4 (450)
C: Chain + Proposed	99.21	38 ± 15	−47.8	−68.1	4.5 (460)

**Table 3:** Three-Room environment over 50 Monte Carlo runs.

Method	Sat [%]	E2E Viol.	E2E Avg. RSSI [dBm]	E2E min RSSI [dBm]	Near-Coll. [%] (Count)
Proposed GP-ATMPC	99.21	38 ± 15	−47.8	−68.1	4.5 (460)
Nominal MPC	99.01	48 ± 20	−47.8	−68.4	4.4 (450)
Heuristic adaptive- margin MPC	98.99	49 ± 25	−47.8	−68.4	4.4 (450)
Reactive CARA	99.79	10 ± 3	−47.0	−66.7	16.6 (1715)

**Table 4:** Warehouse environment over 50 Monte Carlo runs.

Method	Sat [%]	E2E Viol.	E2E Avg. RSSI [dBm]	E2E min RSSI [dBm]	Near-Coll. [%] (Count)
Proposed GP-ATMPC	96.56	218 ± 513	−48.7	−69.1	5.7 (1379)
Nominal MPC	92.74	470 ± 810	−49.2	−69.9	5.6 (1413)
Heuristic adaptive- margin MPC	92.60	478 ± 807	−49.2	−70.0	5.5 (1400)
Reactive CARA	99.97	2 ± 1	−47.3	−65.5	20.0 (4691)

## Data Availability

The original contributions presented in the study are included in the article; further inquiries can be directed to the corresponding author.
